# Developing a diversity, equity and inclusion compass to guide scientific capacity strengthening efforts in Africa

**DOI:** 10.1371/journal.pgph.0002339

**Published:** 2023-12-20

**Authors:** Victoria O. Kasprowicz, Kim Darley Waddilove, Denis Chopera, Sipho Khumalo, Sashin Harilall, Emily B. Wong, Etienne Karita, Eduard J. Sanders, William Kilembe, Simani Gaseitsiwe, Thumbi Ndung’u

**Affiliations:** 1 Africa Health Research Institute, Durban, South Africa; 2 HIV Pathogenesis Programme, The Doris Duke Medical Research Institute, University of KwaZulu-Natal, Durban, South Africa; 3 Ragon Institute of MGH, MIT and Harvard University, Cambridge, Massachusetts, United States of America; 4 Rwanda Zambia Health Research Group, Lusaka and Ndola, Zambia, Kigali, Rwanda; 5 Emory University, Atlanta, GA, United States of America; 6 Kenyan Medical Research Institute-Wellcome Trust Research Programme, Kilifi, Kenya; 7 Nuffield Department of Clinical Medicine, Centre for Clinical Vaccinology and Tropical Medicine, University of Oxford, Headington, United Kingdom; 8 The Aurum Institute, Johannesburg, South Africa; 9 Botswana-Harvard AIDS Institute Partnership, Gaborone, Botswana; 10 Department of Immunology & Infectious Diseases, Harvard T.H. Chan School of Public Health, Boston, Massachusetts, United States of America; 11 Division of Infection and Immunity, University College London, London, United Kingdom; PLOS: Public Library of Science, UNITED STATES

## Abstract

Diversity, equity and inclusion (DEI) in science is vital to improve the scientific process and ensure societal uptake and application of scientific results. DEI challenges include a full spectrum of issues from the lack of, and promotion of, women in science, to the numerous barriers in place that limit representation of African scientists in global scientific efforts. DEI principles in African science remain relatively underdeveloped, with limited engagement and discussion among all stakeholders to ensure that initiatives are relevant to local environments. The Sub-Saharan African Network for TB/HIV research Excellence (SANTHE) is a network of African-led research in HIV, tuberculosis (TB), associated co-morbidities, and emerging pathogens, now based in eight African countries. Our aim, as a scientific capacity strengthening network, was to collaboratively produce a set of DEI guidelines and to represent them visually as a DEI compass. We implemented a consortium-wide survey, focus group discussions and a workshop where we were able to identify the key DEI challenges as viewed by scientists and support staff within the SANTHE network. Three thematic areas were identified: 1. Conquering Biases, 2. Respecting the Needs of a Diverse Workforce (including mental health challenges, physical disability, career stability issues, demands of parenthood, and female-specific challenges), and 3. Promotion of African Science. From this we constructed a compass that included proposed steps to start addressing these issues. The use of the compass metaphor allows ‘re-adjustment/re-positioning’ making this a dynamic output. The compass can become a tool to establish an institution’s DEI priorities and then to progress towards them.

## Background

A culture of Diversity, Equity and Inclusion (DEI) is increasingly recognised as critical to achieve greater scientific productivity and impact, and to address the well-recognised lack of diversity and representation in the scientific workforce [1–3]. Diversity in science is vital to improve the scientific process and ensure societal uptake and application of scientific results [2, 4]. There are many barriers that contribute to suboptimal DEI principles and the underrepresentation of key stakeholders in science and overcoming these challenges may lead to improved science-led practices and policies [5, 6]. While the underrepresentation of women in science, and particularly in scientific leadership, is a global problem, Africa has its own unique challenges associated with this issue [7–12]. Progress in this area was further hampered by the COVID-19 pandemic, in part due to challenges such as additional childcare responsibilities [13]. However, the lack of DEI extends beyond the lack of promotion of women in science, including numerous barriers in place that limit representation of African scientists in global scientific efforts [14–16]. For African science to truly advance both on the African continent and globally, there needs to be greater efforts to support DEI.

The Sub-Saharan African Network for TB/HIV research Excellence (SANTHE) is a network of African-led research in HIV, tuberculosis (TB), associated co-morbidities, and emerging pathogens, now based in eight African countries. SANTHE has four major strategic objectives: to produce world-class scientific research that addresses African health and research priorities in HIV/TB and associated morbidities (including SARS-CoV-2); to increase levels of globally competitive African scientists and research groups by supporting the development of a critical mass of scientists at research sites; to increase knowledge translation and exchange processes to ensure optimal research impact; and to strengthen research ecosystems to facilitate high-quality science and supportive, sustainable, and equitable working environments. Our aim in 2021, as a scientific capacity strengthening network then based in 5 African countries (South Africa, Kenya, Botswana, Rwanda and Zambia), was to collaboratively produce a set of DEI guidelines and to represent them visually as a DEI compass. By guidelines, we mean high-level ideas for changes that could be made to address identified issues within SANTHE. We aim for this compass to be used by SANTHE to support initiatives at its associated institutions. SANTHE has started to implement some tools designed based on the suggested solutions identified in our compass. We aim to then share the process and the resulting compass with others, so they might draw inspiration from it to develop their own compasses as part of a commitment towards improving DEI in scientific research ecosystems in Africa. In addition, we hope to use it as a platform to continue the DEI discussion with others, so we share feedback, learning, and suggestions, to improve our approaches and adjust our compasses on a regular basis. Furthermore, as SANTHE has expanded—adding research sites in Cameroon, Uganda and Zimbabwe- we are now working on an updated compass to include perspectives from all our sites.

## Methods

The Sub-Saharan African Network for TB/HIV Research Excellence (SANTHE) was established in 2015 as a collaborative TB and HIV scientific research and capacity building programme, part of the DELTAS Africa Initiative (https://scienceforafrica.foundation/deltas-africa). At the time these activities- and compass development- took part SANTHE was based at the following sites: the Africa Health Research Institute (AHRI) in Durban South Africa (the lead partner); KEMRI-Wellcome Trust Research Programme (KWTRP) in Kilifi, Kenya; Rwanda Zambia Health Research Group (RZHRG) in Kigali, Rwanda and Lusaka, Zambia; and the Botswana-Harvard AIDS Institute Partnership (BHP) in Gaborone, Botswana. Each site has both junior and senior scientists and support staff supporting SANTHE-associated efforts. SANTHE has since expanded to include the following sites: Centre de Recherche sur les Maladies Emergentes et Re-emergentes (CREMER) in Yaoundé, Cameroon; Uganda-Case Research Collaboration (UCRC) at Makerere University, Kampala, Uganda; and the Collaborative Clinical Research Centre (CCRC) at the University of Zimbabwe, Harare, Zimbabwe. As a consortium we wished to better understand the perceived challenges around DEI by our members and start a collaborative process by which we identified potential solutions or directions in which we should move to make SANTHE more DEI-friendly. Our approach to this was to design and implement a cross-consortium survey, perform focus group discussions and facilitate a SANTHE-wide workshop.

### Consortium-wide survey

**Fig 1:**
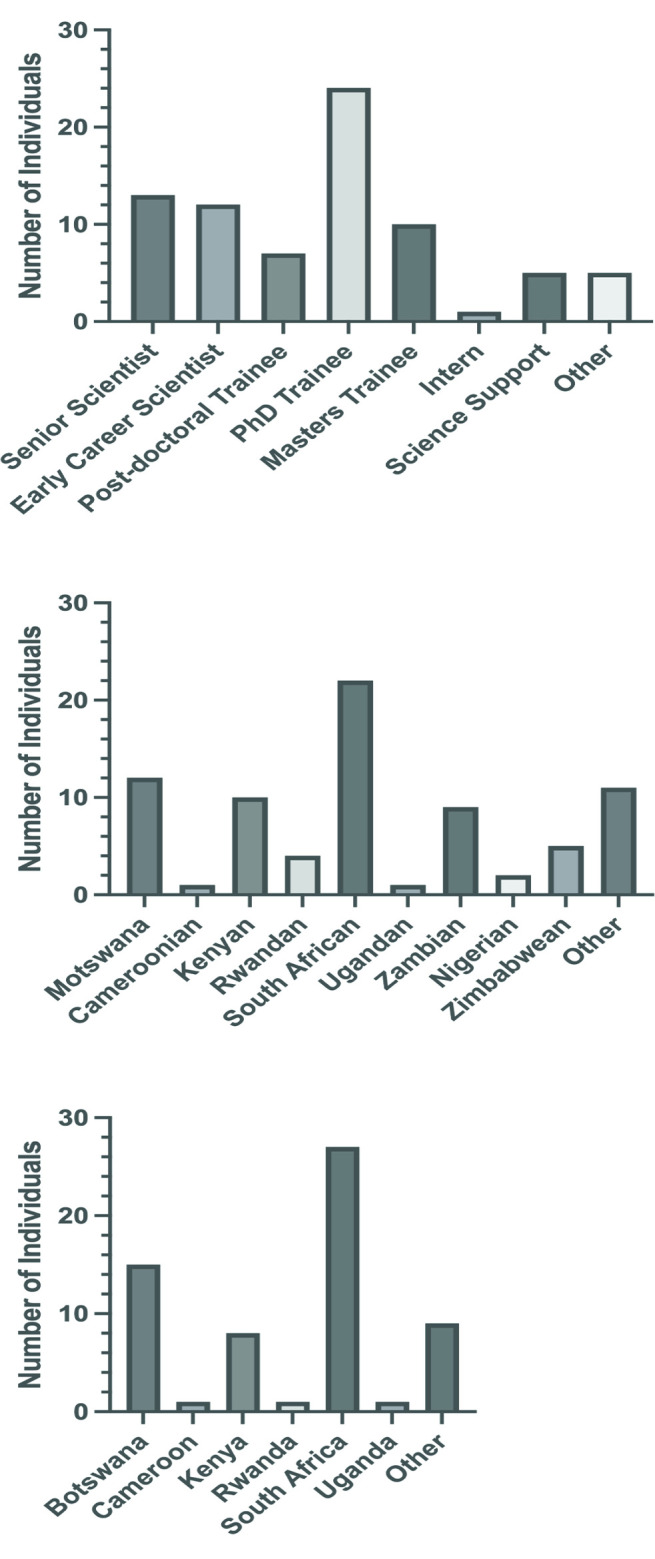
a: Rank of survey participants. Senior scientist was defined as an individual with their own funding support and research team. Early/Mid- Career scientist was defined as an individual who had completed their PhD and post-doctoral training but was not yet fully independent. b: Nationality of survey participants. c: Country of residence of survey participants.

In mid- 2021 we sent out a survey across our consortium to gauge the understanding and experiences of DEI in our members’ scientific careers, targeting both scientific and support staff (survey respondents split by role is displayed in Fig 1A). We received responses from 77 individuals in total, 61% of whom were female (S1 Table). 70% of our survey respondents were black, 17% were white, 9% were of Indian descent, and 4% were other or chose not to identify. Participating individuals ranged from the 18–25 years age group to the 65+ years, however, the vast majority fell into the 26–35 years (35%) and 36–45 years groups (49%). The nationalities of survey respondents are displayed in Fig 1B and their country of residence in Fig 1C.

### Focus group discussions

We then invited our consortium to take part in online focus group discussions (FGDs) using Zoom. The purpose of these small group discussions was to obtain more depth/detail to the issues that were raised in the surveys. Specifically, we wished to 1. Understand perspective of the current DEI landscape in science, 2. Identify specific issues/key concerns, and 3. Begin the discussion on what SANTHE could do to address these issues.

Key questions that the discussions were guided around included: What do we understand by the term ‘equity, diversity and inclusion’?; What do we think about diversity, equity and inclusion in science?; Do we have diversity, equity and inclusion concerns for the future of our careers/those of our colleagues?; Are we able to identify key/top areas of concern?; What can SANTHE do to help?. We also discussed some of the challenges raised through the surveys. We hosted two FGDs, both of which were held online, in part due to the ongoing COVID-19 pandemic and in part due to the geographical distribution of participants. There were 22 participants in the first FGD which took part on 29^th^ January 2021, 68% of whom were female, and 86% of which were Black. There were 4 support staff members, 5 Masters students, 1 graduate intern, 2 senior scientists, 2 early/mid-career scientists, 5 PhD students and 3 post-doctoral trainees. There were 10 participants in the second FGD (13^th^ February 2021), 90% of whom were female. There were 2 support staff members, 3 Masters students, 2 PhD students, 2 post-doctoral trainees and 1 senior scientist. Each FGD lasted just over 60 minutes.

### Online workshop

On 16^th^ April 2021 we held a two-hour online DEI workshop using Zoom. The purpose of this workshop was to discuss potential solutions or directions we could consider taking as a consortium to address the challenges that had been raised through the survey and FGDs. The workshop took part in 3 parts: 1. A brief overview of our efforts to date was provided, 2. We highlighted key issues/concerns raised so far, and 3. We had an open discussion on what steps could be taken to address these issues. All SANTHE scientists and support staff were invited to participate, in addition to interested others at our SANTHE-affiliated partner institutions and other key stakeholders who could potentially suggest innovative solutions or who were in a position to help with the implementation of potential solutions. A total of 91 individuals attended the workshop in which we discussed the identified issues and brainstormed what directions and potential solutions could look like. 63% of attendees were female. 24 were support staff members; 20 senior scientists; 5 mid-career scientists; 17 PhD students; 5 post-doctoral researchers; 2 graduate interns; 12 Masters students; 4 unknown; 1 media; and 1 funder.

### Inclusivity in global research

Additional information regarding the ethical, cultural, and scientific considerations specific to inclusivity in global research is included in the S1 Table.

## Findings

### Overview of survey findings

**Fig 2:**
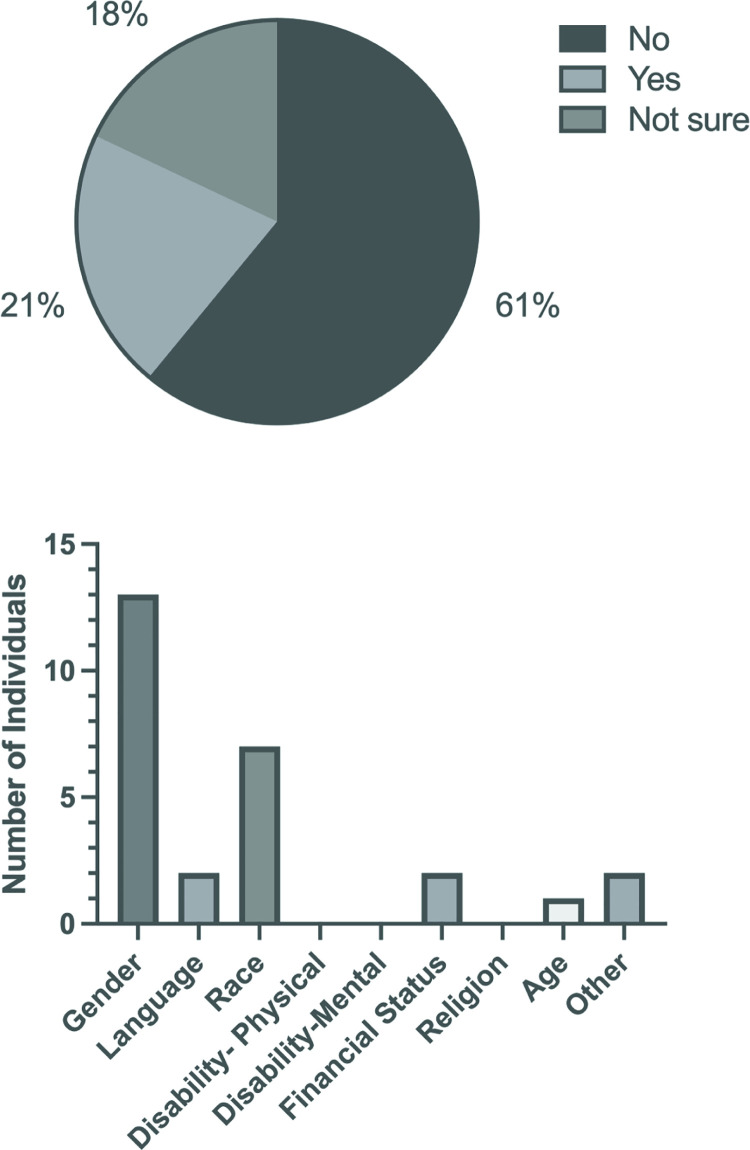
a: Survey responses to the question "do you consider yourself as disadvantaged in the workplace?". b: Survey responses to the question "Why do you consider yourself disadvantaged?".

**Fig 3:**
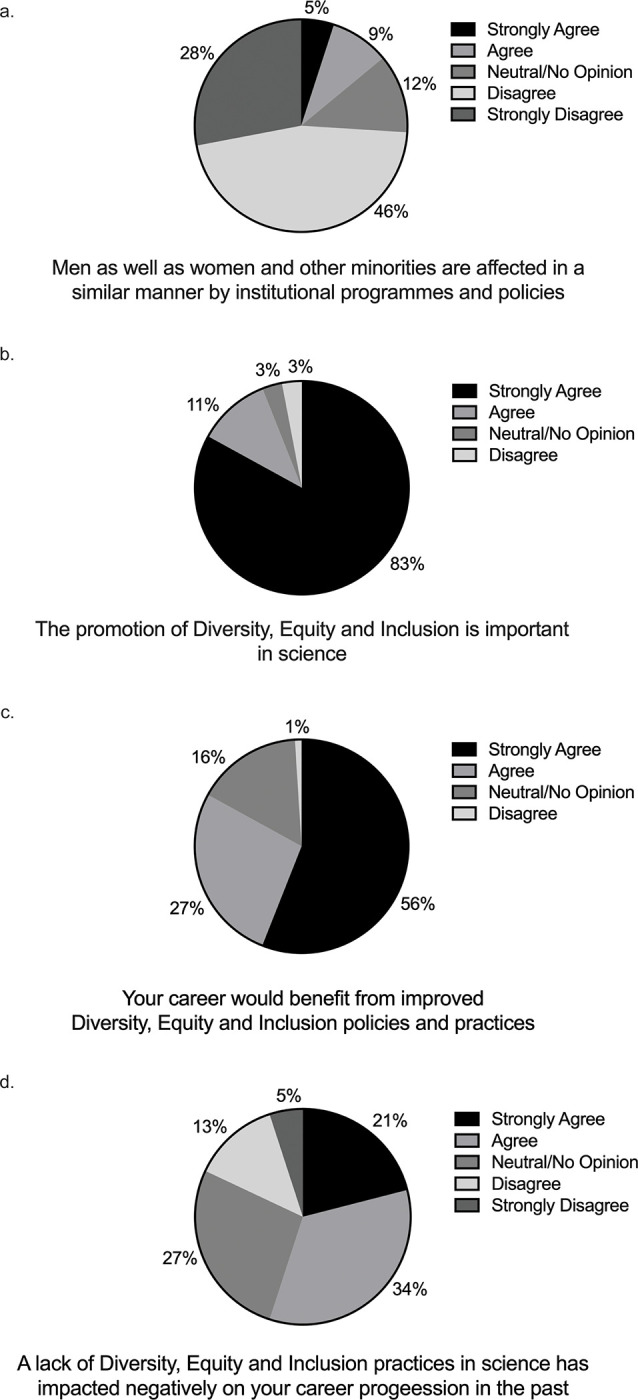
Survey responses—agreement levels with key statements around diversity, equity and inclusion.

**Fig 4:**
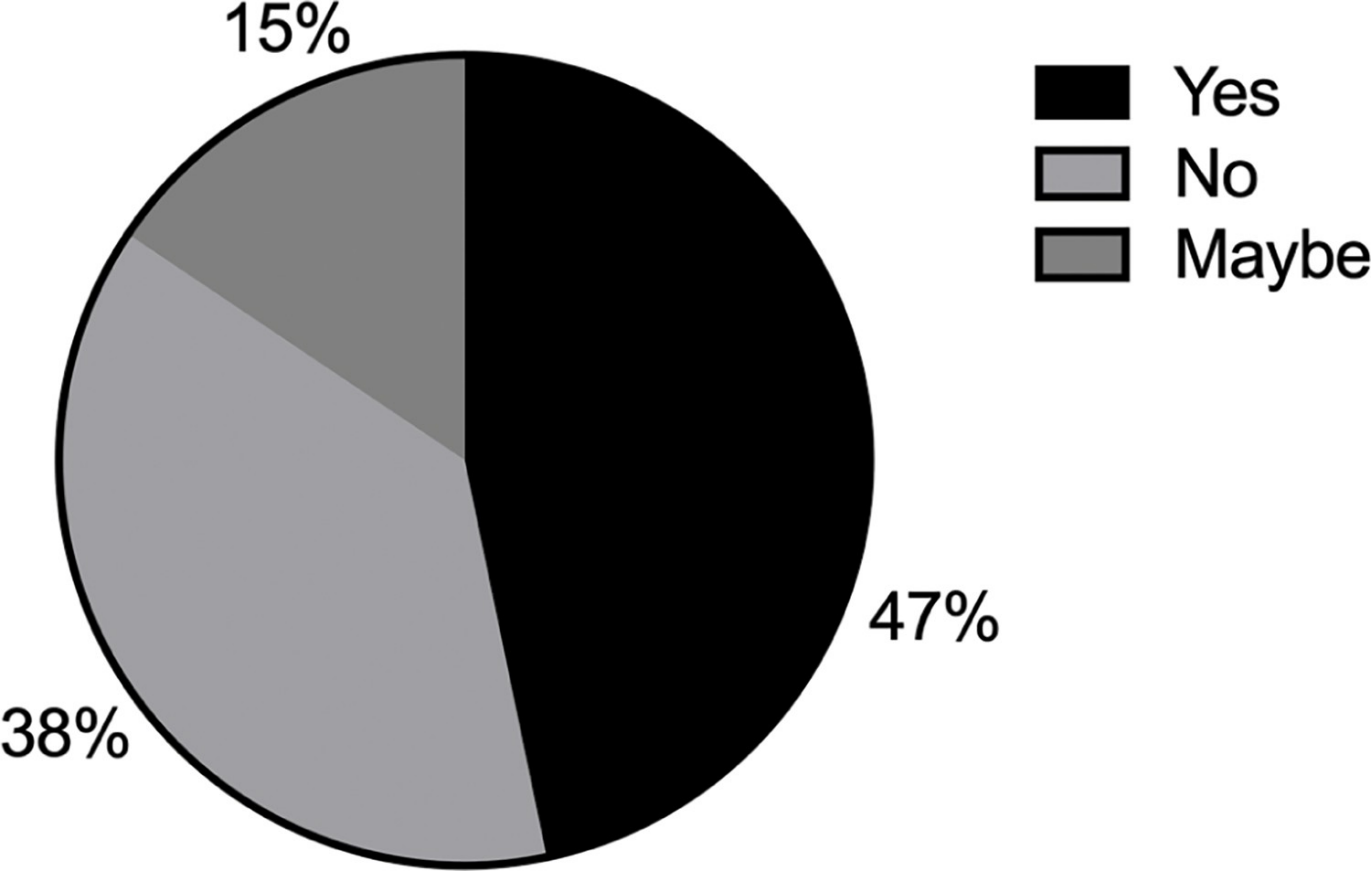
Survey responses to the question "Have you ever witnessed a lack of DEI practices in science negatively impacting on someone else’s career?".

**Fig 5:**
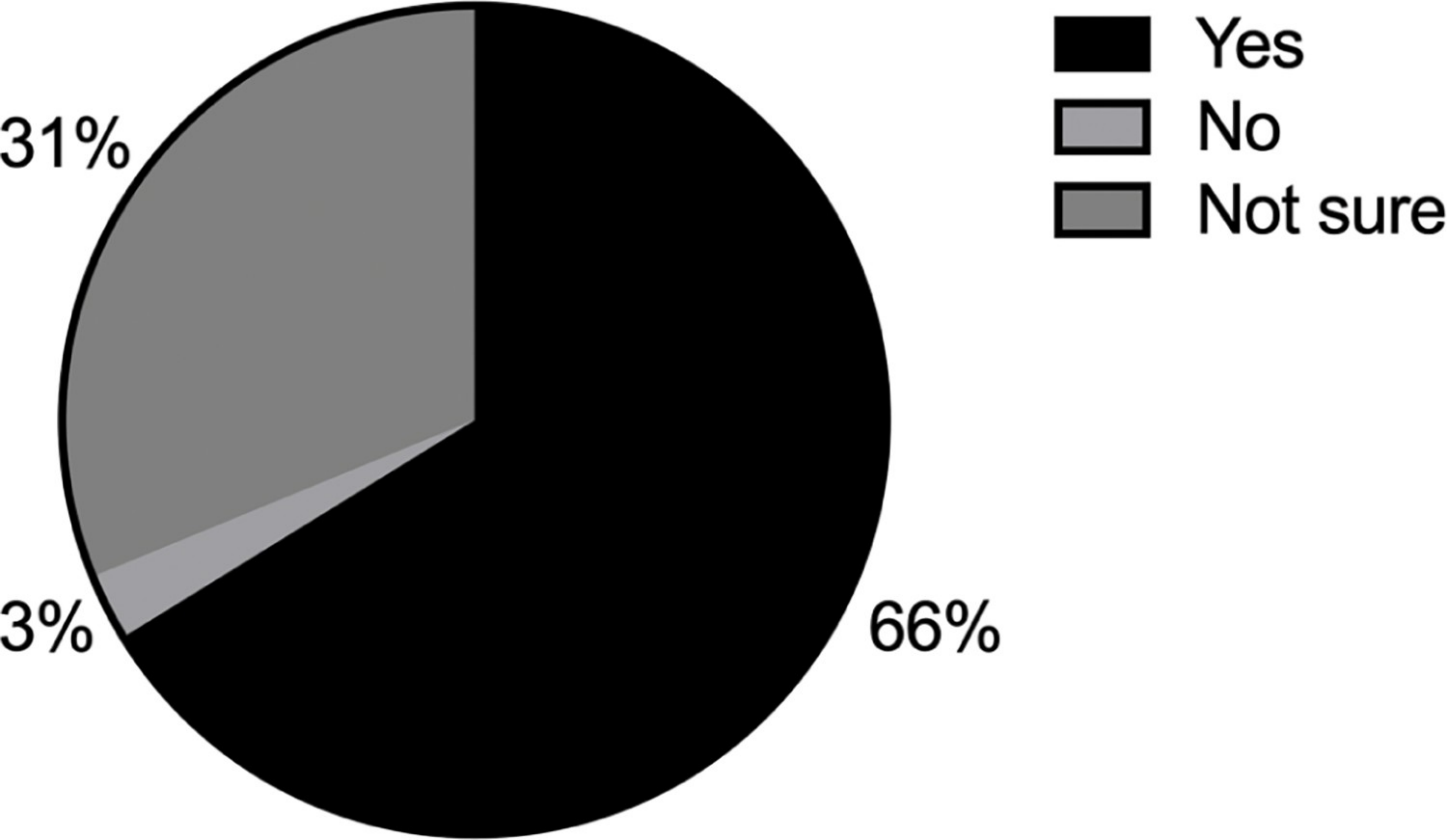
Survey responses when participants were asked: "Do you think that you, as an individual, can do anything to address DEI in your scientific environment?".

21% of those who participated in our survey felt that they were disadvantaged in the workplace, while 18% were unsure (Fig 2A). Those that felt disadvantaged were given the opportunity to indicate the reason as to why they felt that way (more than one option could be selected). Fig 2B reveals that gender was the most popular reason that individuals felt disadvantaged in the workplace, with race receiving the second highest number of selections, and language and financial status coming in with the third greatest number of selections. We next asked survey participants how strongly they agreed or disagreed with pre-selected statements. Fig 3A reveals that most of survey participants felt that different groups are impacted at different levels by the institutional policies in place. There was strong agreement that the promotion of DEI was important in the scientific research ecosystem (Fig 3B). The vast majority agreed or strongly agreed that their own careers would benefit from improved DEI practices and policies (Fig 3C). A significant number agreed or strongly agreed that a lack of DEI practices had impacted negatively on their scientific careers (Fig 3D). Supporting this was the feeling that 47% of survey recipients had witnessed a lack of DEI practices impacting on someone else’s career (Fig 4). We next asked if the survey participants felt that they themselves could do anything to address DEI in their scientific environment and 66% responded positively (Fig 5).

### DEI challenges raised by survey participants

When asked to describe how a lack of DEI practices in science has negatively impacted on your career progression or someone else’s career, responses fell within 4 main topic areas: gender, motherhood, being an African scientist, and race.

Strong sentiments were raised that being a female in science was much harder than being a male and that there was a strong bias against females in the workplace, regardless of qualifications or experience.

“*I noticed that as a woman I am often not recognised even if I am more qualified and have more experience than men*. *Also my views get to be trampled upon by men because some men do not respect women*, *they feel women have a way of blowing things out of proportion*, *even if as a woman you have a genuine concern*, *men are more likely to attach your gender to it*. *“*

ID10

Many felt that this bias against women is clearly reflected in the lack of females in senior leadership positions.

“*While female students are encouraged*, *precious few are promoted to positions of authority or leadership*. *Males protect and defend males*, *even when their competitiveness is counter-productive and inappropriate*. *Functioning within old hierarchical /patriarchal models discourages those of us who wish to work collaboratively with transparency*.*”*

ID29

“*Strong prejudice against the promotion of women into senior academic leadership positions in my institution*.*”*

ID32

“*From a woman’s point of view*, *men tend to climb the scientific ladder or progress smoothly than women*. *As of right now*, *in science*, *the proportion of men occupying leadership or powerful positions is higher than that of women*.*”*

ID36

Although some like ID10 above felt that there was a bias against women because they are seen to be more emotional and less rational, many felt that this bias was driven by the association of the female gender with motherhood.

“*As a female and mother upward trajectory in my career path required additional effort compared to males*. *I had to convince the powers that be that I could lead a research unit after the previous director retired even though my CV attested to my capabilities*. *“*

ID27

“*Although I am from a highly privileged background (nationality*, *academic background*, *etc) even I have had my career impacted by lack of DEI practices in science*. *The easiest to explain are those surrounding maternity leaves (or lack thereof) surrounding the births of my children as well as dips in productivity related to childbearing periods*. *Additionally*, *I am quite certain that perceptions of my science and career prospects have been influenced by people in leadership’s unconscious biases about my gender and race—but these things are harder to be certain about*. *This has also impacted the perception of my contribution to papers and projects when credit/authorship/funding is distributed*.*”*

*ID37*

It was clear that a number of challenges were linked to a lack of resources, infrastructure or policies to optimal support parents in the work-place.

“*As a mother I have many instances where I have been forced to choose between my child and a career advancement opportunity*.*”*

ID3

“*One of my colleagues who is doing MPhil is having a hard time upgrading to PhD because she went on maternity leave and that time is not being recognised*.*”*

ID3

“I *personally know a female scientist that had to drop out from a PhD programme so they can start a family*. *This was due to lack of support and proper childcare assisting services in her institution*, *among other things*.*”*

ID26

“*Pregnant postgraduates are not given support or extension to complete their research*. *Nursing student mothers are denied pumping facilities*.*”*

ID23

”*Science is a demanding career and if you come to it as a mother*, *wife and family person you get drained from trying to balance the two*. *If scientific institutions can be inclusive in their policies*, *like provide proper maternity leave for PhD students*, *have lactation room within institutions etc*.*”*

ID17

“*There’s a tendency of ignoring the needs of working mothers such as their mental health and the agenda of pushing turn-around times becomes the norm*.*”*

ID30

”*I have experienced the frustration of not being able to benefit from the networking and learning opportunities associated with conferences and meetings due to childcare commitments*. *I think conferences/meetings/institutions/employers should consider making provision for childcare to allow meeting attendance*.*”*

ID33

Perceived challenges of being an African scientist operating in a global community were raised by many voices within the SANTHE network. Some comments reflected a feeling that there was a lack of appreciation for African-generated science and scientists.

“*Scientific work conducted in Africa is not considered equal*.*”*

ID9

”*In areas of publication*, *if you include white people as part of authors in your manuscript*, *there are high chances the manuscript will be accepted for publication especially in high impact journals*.*”*

ID37

“*The kind of programme I do is novel in my country but well established in other countries*. *If I had similar access to instruments within the country as other scientists outside the country*, *that would make my progress faster*. *I feel some countries are disadvantaged by acting as specimen collection points only*. *Advanced analysis is conducted in other countries*. *This in my view is a bottleneck on inclusiveness and diversity*. *You cannot easily go with new ideas to outside laboratories because they feel they know more*. *You thus just become a passenger in the old way of thinking and just riding along*.*”*

ID8

This perceived bias against African scientists was also raised around the issue of receiving funding support and other opportunities.

“*Sometimes people who are from the global north have inherent advantages that are unavailable to those in the south*. *They tend to have personal links to officials in funding institutions*.*”*

ID2

“*There is no equal access of opportunities*, *chances are given for the ’correct population’ and other groups are denied or rather excluded*. *They aren’t allowed to partake in sharing of the cake at the high table*.*”*

ID34

Additionally, structural barriers were also raised as a challenge for African scientists in particular issues around obtaining visas and access to key scientific resources such as reagents.

“*Additionally*, *immigration policies in the north put people in the south at disadvantage*. *It is often harder to get entry VISA’s thus limiting opportunities for physical networking*.*“*

ID2

“*Denial of VISA resulted in missing opportunities for meeting attendance*. *“*

ID4

”*In the country where I am*, *every reagent needs to be imported*. *The lead time to access these reagents is long*. *This disadvantages scientific research as certain products needs to be accessed easily*.*“*

ID32

Race was also raised as a key concern with many expressing concerns of bias against black scientists.

“*Non-black scientists in my research group are treated better*. *In terms of project opportunities*, *promotions and financially superior incentives*.*”*

ID22

“*Not being afforded an opportunity to learn and grow in my desired field/passion due to mostly my race then gender has resulted in a slow career growth*.*“*

ID28

The issue of limited senior black scientists in senior roles was viewed as further proof of a systemic bias against black scientists and concerning as also limited role models were perceived to be present.

”*I often feel there are no young black mentors that I could look up to*, *that have walked my path*. *Often there are no mentors that are faced with discrimination of academic citizenship like I have*. *Maybe they are there and are not open about their journey*.*“*

ID12

“*Lack of representation from advisors and supervisors who were from a different background e*.*g*. *culturally*, *negatively affected my career earlier in my postgraduate studies*. *This affected our communication and work engagements because of racial stereotypes that existed and were generally expressed*. *It is important to have people that look like you and are from a similar background as you when you are still channelling and carving your path as a young scientist*. *These relationships help you navigate things better and even enable you to face the global science community with confidence*.*”*

ID26

Another point captured through the survey was that token gestures are often not enough and that institutions/organizations need to provide depth to the action they take around DEI.

“*I believe equality well understood but inclusivity is often misunderstood*. *Lack of inclusivity can be as simple as not giving a person or group of people a voice in the room*.*”*

ID15

“*Institutions policies including equity seems to be driven more towards operational staff rather than science people*. *Recruitment or opportunities don’t really take a stance on DEI—meaning there is no active plan or description*.*”*

ID22

”*One observation to share that may be unexpected is to have seen what appears to have been a positive DEI practice*, *e*.*g*. *hiring a person from a previously disadvantaged community*, *but very inadequate support and capacity development for the person*.*”*

ID20

### Suggested actions to support DEI

There were a number of suggestions or comments around what individual action could be taken to help improve DEI. A number of individuals commented about future action they could take including thinking carefully when hiring new staff members to make sure e.g. gender is balanced, and for advocating for DEI improvements in policies and resources decided at institutional level.

“*By providing an enabling environment within my organisation that encourages individuals disadvantaged by social circumstance to have equal opportunities like everyone else*. *In my environment this is mostly a gender issue i*.*e*. *women vs men*. *I can influence employment to ensure equal distribution of men and women in the institution*. *When training opportunities arise*, *deliberate organisation policy to equitably distribute these opportunities among capable males and females*. *As a way of providing an enabling environment*, *I’d advocate for other support for women particularly during child bearing period and nursing infants/babies; this will be support that enables females to achieve their career goals e*.*g*. *providing an allowance for child care/maid*.*”*

ID2

“*When I run my lab I can put diversity*, *equity and inclusion as a top value of my lab and use this for who I recruit to my lab*. *We can take extra time to listen to each-others ideas*, *discuss EDI topics among each other*, *and respectfully call out each-others’ implicit biases and mistakes and improve from that*. *In my teaching*, *I analyse every statement I make effort to try to ensure it has the intent of fostering inclusion in my class*, *not exclusion*.*“*

ID7

“*Yes*, *I think we all have to constantly educate ourselves on this topic*, *consciously read the writing and thinking of women and non-white cis-men and work consciously to combat our conscious and unconscious biases in all our academic activities (leadership*, *mentorship*, *sponsorship*, *writing*, *reviewing*, *editing*, *etc)*. *We should speak up when necessary and strive to make our actions at minimum equitable and preferably actually in favour of transformation (in the South African sense)*.*“*

ID40

Others commented about efforts they had already made emphasising their awareness and support for improving DEI in African science.

”*Yes*, *and I have*. *As a supervisor and director of my research unit I have always tried to address previous inequalities and to be understanding of the different needs of various groups*. *For examples I have tried to appoint women and to understand the challenges of home/work balancing act*. *Also I am acutely aware of the challenges of previously disadvantaged individuals in terms of resources*, *living conditions and home environment*. *Therefore I have always tried to support students and staff both morally and financially*, *when I had the resources*. *I have always tried to address imbalances and to give people the opportunity to fulfil their full potential*. *I have always been encouraging and have tried to inspire*.*“*

ID31

Members of the network shared some initial suggestions of what could also be implemented at an institutional level to support DEI. Some commented on improving policies around maternity leave.

“*Yes*. *My employer can start by giving 100% when one is on maternity leave instead of 60% even though no replacement is ever hired*.*“*

ID3

However, many comments were linked to creating a DEI-friendly culture, with effective training and dialogues taking place around this topic.

**“***Make others more aware of any biases they may hold and how these affect early career scientists*, *talk about the issues at large more often*, *training seminars*, *sensitivity training*.*“*

ID17

”*Make this a priority—ensure that genuine training is done*, *internalised*, *and acted upon by all faculty and leadership*. *Encourage a true culture of equity and transformation that truly celebrates diversity*. *Consciously work against paternalism and side-lining women and people from non-White racial groups*. *Clear and progressive maternity and care-giving policies and consideration when it comes to promotion/evaluation*.*”*

*ID45*

”*Improved consultation with marginalised students*, *trainees and others who are being marginalised to seek meaningful approaches to strengthen DEI practices and approaches to realise improved accountability for strategies approved for strengthened practices*.*”*

*ID22*

“*Create an environment where workers feel their diversity is celebrated and that equity and inclusion are not simply writings in documents but the daily experiences by everyone irrespective of their cadres or levels in the organisation*.*”*

ID18

”*Can have a whistle blowing policy that allows individuals report any issues that are affecting inclusivity or equity*.*”*

*ID4*

Importantly, there was feeling raised that institutions could be doing more to improve research continent on the continent, which in then could help facilitate enhancing the quality and perception of African science in the global scientific community.

“*By being more proactive in advancing research capacity*.*”*

*ID9*

### Key feedback from the FGDs

The discussions from the FGDs built on the information gained from the surveys. Three themes started to emerge from the FGD conversations. The first was around this idea of “bias” in the workplace- either conscious or unconscious. For example, the strong bias against mothers was raised, the lack of female role models and the need to change the patriarchal structure in science. In addition to gender bias, cultural bias was raised and the perception that science generated from Africa was not respected.

The second theme that emerged is the need for science/institutions to be more flexible in their approach to better support DEI through greater understanding of the needs of their workforce. For example, concerns were raised about how individuals on the academic career track are treated like trainees and are not provided employee-associated benefits. Individuals also felt that a lack of ‘respect’ was given to them as they are treated like students and there was a strong feeling of a lack of ‘job’ security, impacting on mental health. Although this is a global challenge, it is a greater problem in the African setting where many scientists have increased family responsibilities at a younger age. The final theme that started to emerge was around perceived specific barriers for African scientists. For example, funding was perceived to be skewed with scientists in Africa having limited opportunities. Comments were also made regarding other DEI barriers in e.g. *Nature* journals’ high publication fee that is well beyond the means of most African researchers, and the feeling that everyone should be able to submit their science regardless of how much it costs to publish. Access to journals was also reported to be a key challenge as many journals/journal articles are still not open access.

These three thematic areas are summarized below with the key issues that were raised under these themes from both the survey and FGDs:

1. Conquering Biases—(including sexual orientation bias, cultural gender-stereotyping bias, race preference, male bias, ageism, imposter syndrome, anti-motherhood sentiments, and socio-economic bias)
2. Respecting the Needs of a Diverse Workforce–(including mental health challenges, physical disability, career stability issues, demands of parenthood, and female-specific challenges)
3. Promotion of African Science—(including a lack of appreciation of African-generated science and scientists, preferential treatment of northern-based scientists and institutions e.g. biased reviews of grants/publications, and structural barriers in science that prevent true diversity such as high publication costs).

**Fig 6:**
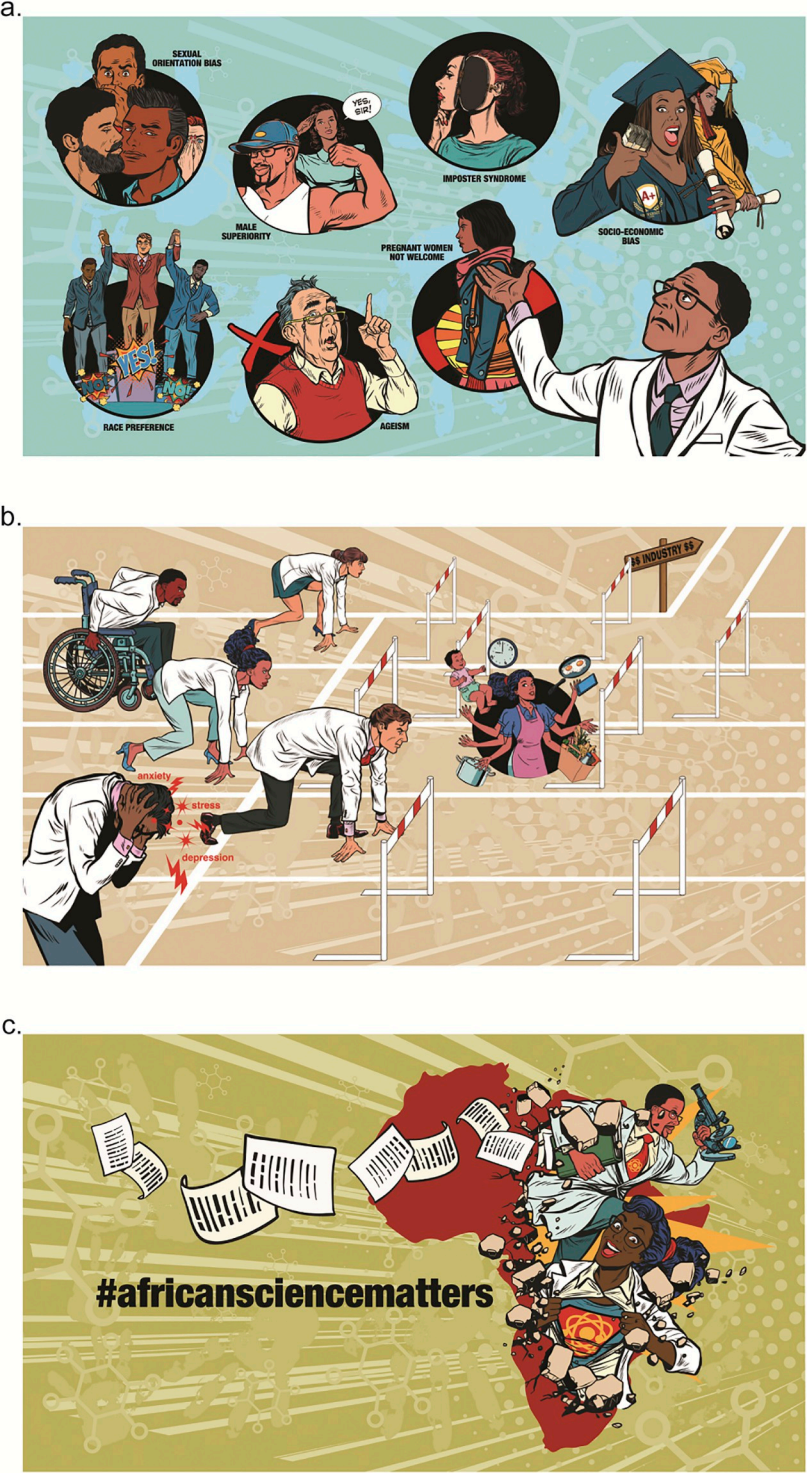
Visual representation of the thematic areas and challenges identified. a: Respecting the needs of a diverse workforce. B: Conquering Biases. C: Promotion of African Science.

The issues included under these themes are highlighted in Fig 6A–6C that were generated specifically for this project and then used in the subsequent workshop to communicate key examples of some of the challenges we face as a consortium.

### Key feedback from the DEI workshop

*The three* identified thematic areas were presented at the workshop and the subsequent discussions in the workshop were guided towards identifying and brainstorming what could be done to help address the identified issues—looking at both identifying the direction that could be taken and then the possible solutions.

### Conquering biases

The directions and potential solutions that were identified for the theme ‘conquering biases’ included:

1. Greater representation of minorities in leadership positions. Potential solutions: promote diversity in selection panels; leadership development training programmes to grow competitive future leaders; and programmes to engage with disadvantaged youth, including females, to encourage interest in science.
2. Minority specific mentorship programmes. Potential solutions: Set up support groups e.g. Women in Science; and assigned supplementary mentors as role models.
3. Remove the bias in scientific project review and funding processes. Potential solutions: embed diversity in scientific review committees; and anonymise application material.
4. Being intentional in promoting and supporting minorities in science. Potential solution: acknowledge and promote minority role models through regular communication campaigns.
5. Actively tackle bias in the workplace. Potential solutions: Unconscious bias training; conduct team building events; and acknowledge internationally and locally recognised days and events that celebrate diversity.
6. Actively acknowledge DEI in the work environment. Potential solution: put institutional (and representative) DEI committees in place to provide recommendations and support.

### Respecting the needs of a diverse workforce

The directions and potential solutions that were identified for the theme ‘respecting the needs of a diverse workforce’ included:

1. Allocate specific funding to support DEI. Possible solutions: fellowship top-ups to cover trainees on paid parental leave; travel awards to cover additional costs associated with attending work/training for parents and disabled scientists; and career re-entry grants.
2. Improve support for working parents. Possible solutions: flexible working options (hours and location); embrace technology to support flexible working; improved paternity and maternity leave; and childcare facilities at workplaces.
3. Introduce mechanisms/procedures for addressing DEI complaints and concerns. Possible solutions: create platforms for anonymous reporting; allow for confidential feedback in trainee progress reporting and evaluations; and put DEI committees in place to provide recommendations and support.
4. Improve support for employees engaged in research as a mechanism to maintain minority engagement in academia. Possible solution: offer protected time to allow employees to focus on their research.
5. Actively promote good practices in DEI in the work environment. Possible solutions: conduct institutional DEI audits including all laboratories/research groups; use audit findings to optimise policies and procedures; and provide training to supervisors and management to support effective implementation of DEI solutions at laboratory and institutional levels.
6. Investigate options to reduce career stability associated stress. Possible solutions: regularly assess scientists’ health and wellbeing; integrate mental health support in current support structures e.g. thesis advisory committees; establish and support peer support groups; and encourage a culture of good health practices.

### Promotion of African science

The directions and potential solutions that were identified for the theme ‘promotion of African science’ included:

1. Africans to take on more leadership roles in scientific and capacity strengthening collaborative efforts. Potential solutions: leadership training/coaching approaches; high-quality mentorships and sponsorship of African scientists; strengthen south-south partnerships; promote equitable north-south partnerships; and the development of next generation of African leaders.
2. More- and new- sources of funding required for African-led research and training. Potential solutions: identify and approach potential philanthropists and corporates for financial support including those based in Africa; enhance communications/build relationships with governments to improve funding opportunities; undertake organised, structured fundraising drive; and training on how to secure research funding.
3. Evaluate scientific structures that contribute to the inhibition of African science. Potential solutions: audit and advocate for systemic changes e.g. high publication costs; and disrupt biases that work against African scientific institutions and African-based scientists.
4. Active promotion of African science and scientists. Potential solutions: promote the full spectrum of African scientific research from Astronomy to Zoology and the scientists involved in this research; support existing initiatives that popularise African scientists; and key communications training for scientists including traditional (media interviews) and social media (Science Twitter).

Our discussions also highlighted key activities that can be done at an individual level. These were: practice good citizenship for DEI; attend DEI-related training; advocate for awareness and change: act in a DEI appropriate manner; acknowledge diversity in all its forms e.g. cultural and religious holidays; and participate in DEI-focused projects e.g. schools‘ engagement and mentorship programmes.

**Fig 7:**
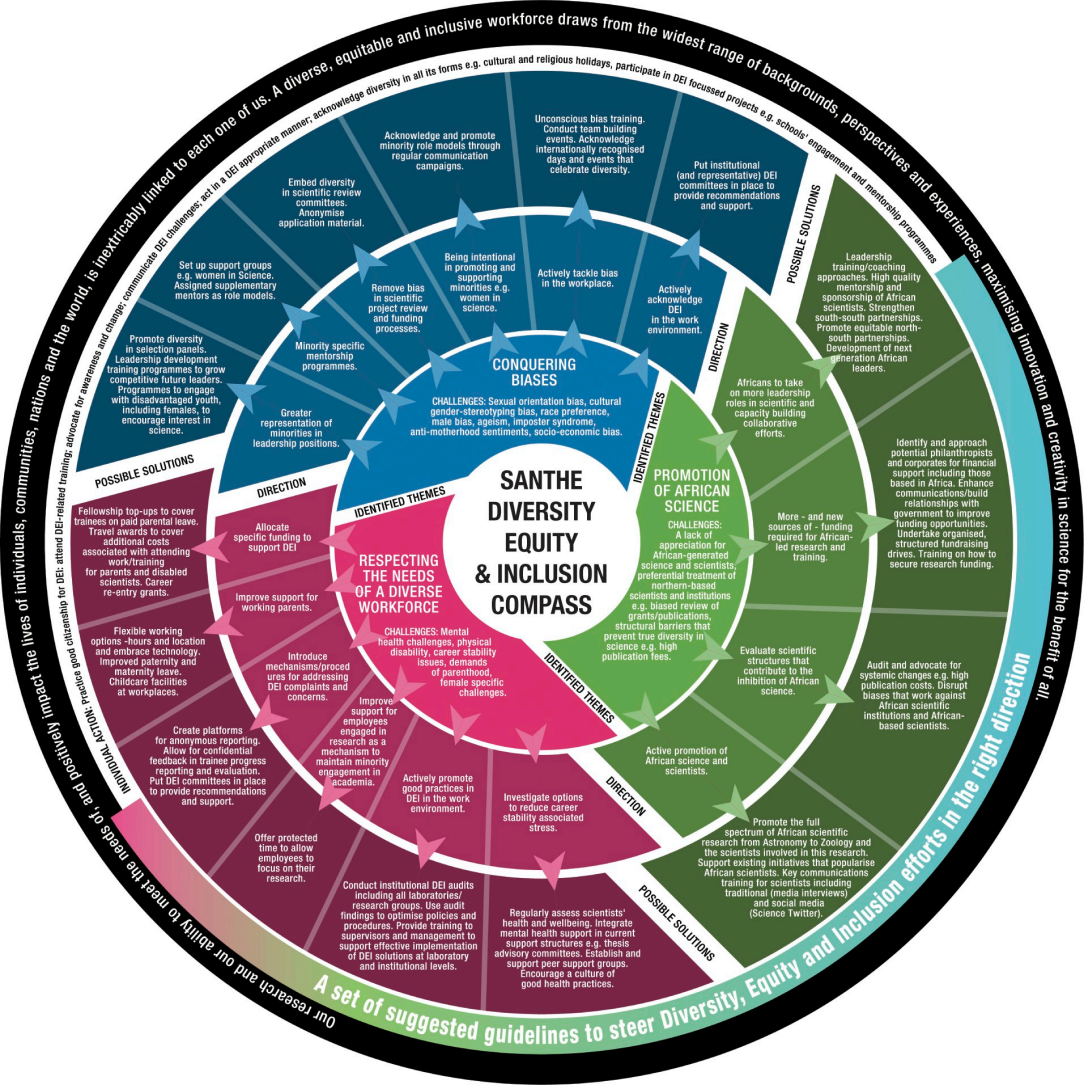
SANTHE diversity and inclusion compass.

The workshop conversations helped guide the development of the content of the first draft of our DEI compass. Drafts of the compass were shared internally and externally, and the feedback was incorporated to create the latest version (Fig 7). Surrounding our compass is our guiding belief: *Our research and our ability to meet the needs of*, *and positively impact the lives of individuals*, *communities*, *nations*, *and the world*, *is inextricably linked to each one of us*. *A diverse*, *equitable and inclusive workforce draws from the widest range of backgrounds*, *perspectives and experiences*, *maximising innovation and creativity in science for the benefit of all*.

## Discussion

Globally there are DEI challenges in science [1–3, 12]. Much of the global discussion has focused on the promotion of female scientists and under-representation of certain communities and ethnic and racial groups [17–19]. DEI principles in Africa science also remain relatively underdeveloped, with limited engagement and discussion among all stakeholders to ensure that initiatives are relevant to local environments [7, 10, 11, 16]. Members of our consortium were motivated to take part in this effort reported here and demonstrated enthusiasm to air their perspectives and contribute to solutions for the challenges identified. We identified three (somewhat overlapping) themes under which most of the issues/challenges raised by members of our consortium fell and around which our current compass is based: conquering biases, respecting the needs of a diverse workforce, and the promotion of African science.

To start conquering biases we have taken steps to introduce training around unconscious and conscious bias. We have also introduced some tools to help us better respect the needs of a diverse workforce. For example, SANTHE has now introduced Care-Giver support awards that provide funding support to assist individuals who act as caregivers to attend scientific meetings or key trainings. These awards cover the additional costs incurred by the caregiver being away or help cover costs for e.g. for parents to bring their children with them. We have also introduced our Fellowship Interruption Awards which help provide additional funds to cover a break within the Fellowship period e.g. perhaps due to prolonged ill health or maternity/paternity leave. A major issue that emerged from our efforts was the challenge of mental health support for all our scientists. Our consortium–and particularly the PhD and post-doctoral trainees–had very strong feelings about this key challenge and agreed that more needed to be done to support scientists in what is probably universally acknowledged, not to be the easiest of career paths. The stress and uncertainty of the scientific career pathway, the lack of respect of being perceived as a ‘student’ even though you have been working for many years, the lack of sufficient locally-based role models and mentors, and the inherent difficulties associated with performing science in Africa were all felt to be major contributors to mental health challenges in our consortium [20–22]. SANTHE has started to take steps to assist in this area e.g. provided a mini-gender equity award to doctoral trainees in our network to support their own mental health campaign and going forward at a network level we will be providing mental health training and support. Recent events have further highlighted the need for support of this kind with the stresses of the COVID-19 pandemic and specific local challenges such as the 2021 riots and 2022 floods at our Durban, South Africa site.

There are numerous barriers that limit the representation of African scientists in global scientific efforts and contribute to the continued colonisation of research involving the African continent [6]. This theme of our compass is perhaps the most challenging for us to address (primarily because it requires additional resources that are not readily available in low and middle income settings), and while we intend to do as much as we can at a consortium and individual level–we also hope that by promoting our compass and our efforts we may also inspire those in external power positions to make key changes. There is need to promote and normalise African excellence. Promoting DEI in African institutions ensures greater access and representation in research, providing opportunities for talent to emerge in Africa, which in turn yields excellence. A key challenge is a lack of visibility of African scientists and their science in the global context. Even as some funding bodies are reflecting on their approach to supporting research on the African continent, there is a lack of knowledge around what science is currently being performed and who leads it. This lack of engagement with African scientists needs to be tackled, so that there is an increased awareness of who does what and where in Africa by both funders and other scientific stakeholders. Our efforts have highlighted the issue of perceived preferential treatment of northern-based scientists and institutions and this is also reflected in the literature. One way to help disrupt potential biases like this is to try and rebrand African science and her scientists, increasing the visibility of what science looks like in Africa and the importance of the research agenda and activities on the continent being led by Africa. In addition, structural barriers also exist that prevent true diversity in science such as high publication fees and publishing structures that continue to favour Northern-based researchers, challenges obtaining visas for travel to key events (e.g. IAS 2022 Montreal and Health Systems Research 2022 in Bogata that lead to the social media slogan #visaapartheid), and language barriers to participating in an anglophone dominated industry [14, 15, 23, 24]. We identified a range of possible solutions that can be implemented to help contribute to the elimination or reduction in the above challenges and we hope that our compass, and hopefully future compasses that are developed may suggest novel approaches to do this or share best practices so that others can also benefit.

We acknowledge some limitations with our efforts reported here. Firstly, given the relatively limited number of respondents we did not narrow down the analysis of the DEI issues brought up by the respondents based on their gender, nationality and race. Given the diversity of the cultures of the respondents, it could be that there are country/regional or race specific DEI issues. Secondly, we wish to highlight our awareness of other DEI challenges that are not outlined or highlighted in depth here. For example, we are aware of the importance of the recognition of lesbian, gay, bisexual, and transgender (LGBT) populations. However, these issues were not raised as one of the majority priority areas by individuals in our network who took part in the efforts described here, and we wished to accurately reflect their current perspectives at this point in time. We acknowledge that these perspectives may change over time, and may change when the sample of respondents increases or when new individuals join our network, hence the importance of routinely updating the compass, and in general promoting good DEI practices in all our activities and approaches including the language we use. Since this compass was created we have added new research sites (in Cameroon, Uganda and Zimbabwe) to the SANTHE network and so are now taking steps to renew our compass to also reflect the new voices from our consortium.

We view the SANTHE compass as a visual commitment to improving DEI within our network. It is not a simple tool—with several layers contributing to its complexity. This is in part due to the granularity of people’s experiences and the specificity of suggestions, which we believe help move away from action-less platitudes which can be a problem in this space. We aim to update our compass routinely, based on feedback and progress, and ideally hope to add best practices as they develop, to make sure that DEI is embedded in all we do. One challenge we have is that SANTHE is a network and has no direct control over institutional practices and policies at the institutions that are part of SANTHE. However, we believe that by sharing the compass both locally and further afield, advocating for DEI, and contributing to the DEI discussion and landscape, that our efforts will have an impact beyond SANTHE. What was interesting to see was the interest in DEI displayed by individuals within the SANTHE network, with many clearly feeling that they had power to do more at an individual level- both in terms of advocacy and action- to support this key area. We hope that this project will inspire others to perhaps develop their own DEI compasses, and we can continue to learn and share from one another when implementing new approaches in this arena. The use of the compass metaphor allows ‘re-adjustment/re-positioning’ making this a dynamic output. We encourage institutions to define annual actions aligned with the compass and to assess their achievements against the goals at least annually and hopefully take each goal to the next level or readjust it as necessary. Thus, the compass can become a tool to establish an institution’s DEI priorities and then to progress towards them.

## Supporting information

S1 Table Details of individuals who completed the survey. (DOCX)
