# Supplementary material for: Developing a diversity, equity and inclusion compass to guide scientific capacity strengthening efforts in Africa
Source: PLOS Glob Public Health. 2023 Dec 20;3(12):e0002339. doi: 10.1371/journal.pgph.0002339 (PMC10732426; doi:10.1371/journal.pgph.0002339)
Supplement: S1 Table — (DOCX) [file pgph.0002339.s001.docx]

S1 Table. Details of individuals who completed the survey.
